# A New Centralized Clustering Algorithm for Wireless Sensor Networks

**DOI:** 10.3390/s19204391

**Published:** 2019-10-11

**Authors:** Juan-Carlos Cuevas-Martinez, Antonio-Jesus Yuste-Delgado, Antonio-Jose Leon-Sanchez, Antonio-Jose Saez-Castillo, Alicia Triviño-Cabrera

**Affiliations:** 1 Department of Telecommunication Engineering, Universidad de Jaén, 23100 Linares, Spain; jccuevas@ujaen.es (J.-C.C.-M.); ajls0006@red.ujaen.es (A.-J.L.-S.); 2 Department of Statistics and Operational Research, Universidad de Jaén, 23100 Linares, Spain; ajsaez@ujaen.es; 3 Department of Electrical Engineering, Universidad de Málaga, 29071 Málaga, Spain; atc@uma.es

**Keywords:** wireless sensor networks, clustering, fuzzy system

## Abstract

Clustering is presently one of the main routing techniques employed in randomly deployed wireless sensor networks. This paper describes a novel centralized unequal clustering method for wireless sensor networks. The goals of the algorithm are to prolong the network lifetime and increase the reliability of the network while not compromising the data transmission. In the proposed method, the Base Station decides on the cluster heads according to the best scores obtained from a Type-2 Fuzzy system. The input parameters of the fuzzy system are estimated by the base station or gathered from the network with a careful design that reduces the control message exchange. The whole network is controlled by the base station in a rounds-based schedule that alternates rounds when the base station elects cluster heads, with other rounds in which the cluster heads previously elected, gather data from their contributing nodes and forward them to the base station. The setting of the number of rounds in which the Base Station keeps the same set of cluster heads is another contribution of the present paper. The results show significant improvements achieved by the proposal when compared to other current clustering methods.

## 1. Introduction

Wireless Sensor Networks (WSN) are composed of multiple nodes, which are deployed randomly in most situations. Although there are some commercial solutions for this kind of network in e-health, smart grids or surveillance [1], there are still some technological aspects that could be addressed to improve their performance. Presently, their main failing is the durability of the network in an autonomous and reliable way. For this reason, there are several proposals to reduce the energy consumption of the nodes while it operates correctly. This energy consumption is mainly caused by the data gathering and the data transmission.

The formation of groups or clusters in this kind of network helps in the reduction of the energy dissipation during the data transmission especially when considering scalability and robustness [2]. As a consequence, the network autonomous lifetime (without the need for replacing the nodes’ batteries) is prolonged when clustering is conveniently applied. In a cluster, there is a node, referred to as the Cluster Head (CH), which is in charge for the communication with other CHs or with the Base Station. It recollects the data from the nodes belonging to its cluster, it aggregates this information and then proceeds to send it to the Base Station (BS). The base station or sink decides the actions to take according to the data.

The decision about which nodes are the CHs can be taken centrally by the BS or in a distributed mode by the sensor nodes. Centralized clustering algorithms demonstrated their ability to have an optimal behaviour in comparison with their distributed counterparts as more information can be used to create the clusters appropriately [3].

Another important issue related to clustering is how long the cluster structure is going to be maintained. The initial proposals on clustering algorithms set that the clusters should be periodically formed. In particular, they opted for deciding about the CHs in each round. A round is the interval of time into which the network lifetime is divided. In a round, the inter-cluster communication, the data fusion/aggregation and the intra-cluster communication sequentially take place to send the measurements to the BS. Therefore, the first proposals (e.g., LEACH-C [4]) included the determination of the CHs at the beginning of each round.

An alternative and novel approach consists of including the execution of the selection of the CHs only in some rounds. The skip stands for the number of rounds in which the cluster heads will be kept [5]. The work in [5] sets the skip as a constant parameter and equal to 2. However, the appropriate value for this parameter is related to the network density, the nodes’ positions and the traffic pattern.

In the present paper, we propose a novel centralized clustering algorithm that decides the cluster heads while it sets an optimized skip. The algorithm, named as “Centralized Skip Based Algorithm” or CSBA, is based on two hypotheses. The first hypothesis, or H1, relies on the fact that when a CH is conveniently elected, maintaining it during some rounds will make the network lifetime longer as the control information (and the consumption related to the transmission and reception of these frames) does not need to be exchanged. However, the second hypothesis on which CSBA is based, or H2, is that keeping a node as a CH indefinitely degrades the network performance in terms of reliability, that is, not all the measurements are collected from the nodes. This happens because the node acting as CH will receive information from the nodes which are ascribed to it. As a consequence, the energetic dissipation of this node increases, especially when this operation is prolonged over several rounds. The measurements that should be collected by this node cannot be guaranteed to be transmitted. In our proposal, the selection of the cluster heads is modelled by a Type-2 Fuzzy system incorporated into the Base Station. Then, the skip is set by an experimental evaluation of the network performance in terms of reliability and durability. The implementation of the technique demonstrates the extension of the network lifetime when compared with other clustering algorithms. Therefore, this paper presents the following contributions:

- The demonstration of the benefits obtained when a skip is set. As described in hypothesis H1, a carefully selected skip value significantly improves the lifetime of a WSN.The paper also verifies that nodes elected as CHs cannot keep this role for an undetermined period because they will deplete their batteries prematurely and degrade network reliability. This last behaviour is compliant with hypothesis H2.
- The definition and validation of a Type-2 fuzzy system to manage the uncertainty of the network information when deciding the CHs with reduced control messages.
- The proposed algorithm was tested in a wide set of scenarios and compared with a significant number of clustering algorithms.

The rest of the paper is structured as follows. The next section presents a review and a classification on the algorithms used to select the CHs. Section 3 describes the proposed fuzzy system, giving a detailed explanation about how this system was designed. Section 4 describes the simulation results obtained to evaluate the proposed algorithm. Finally, Section 5 includes the conclusions and the future research guidelines.

## 2. Related Work

**Figure 1:**
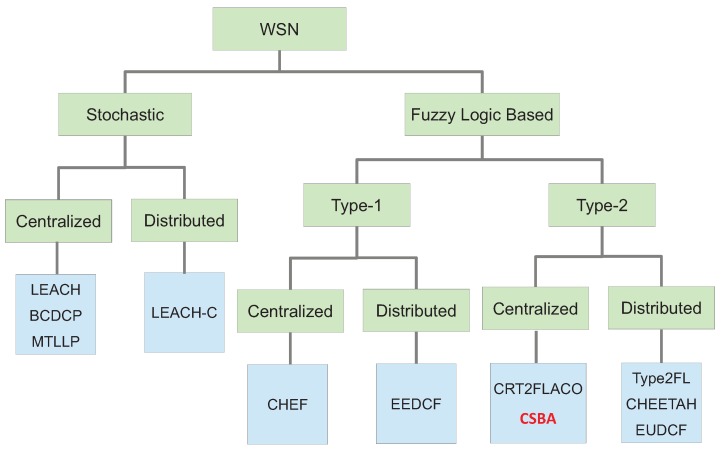
Simple taxonomy of clustering in wireless sensor networks.

Clustering in wireless sensors networks has recently been the objective of deep research [6] because of its suitability for this type of network as it was stated by Liu in [2]. Moreover, clustering allows for many other important performance improvements in a WSN, such as scalability, reduction of the traffic load, decrement of the energy consumption, load balancing and fault tolerance. In [7] the taxonomy presented for cluster head election establishes three categories: preset, random and attribute-based. However, preset election relies on a predefined node deployment that usually uses a previous selection based on an attribute-based algorithm so this category can be included in the last one. Therefore, in general terms, we can state that the method to select the CHs can be classified in two main groups: stochastic and attribute-based. Moreover, taking into account recent publications, attribute-based methods are commonly based on computational intelligence. Specifically, they use a fuzzy logic system because of its simplicity. Alternatively, stochastic protocols are those in which nodes convert into CH basing on a certain random value or a determined mathematical function related with a value provided by the nodes (energy, distance to the base station, etc.). This value is then compared with another random value to finally decide about being CH or not. In Fuzzy Logic Based algorithms, the mathematical function is replaced by computational intelligence. This taxonomy is presented in Figure 1.

Additionally, it is common that the methods to elect CHs are also classified into centralized and distributed ones [7]. In centralized methods, a central node, called base station or sink, which usually has improved computation capabilities, accomplishes all the process and calculation to select the CH in the network based on information gathered from all the nodes. In contrast, in distributed methods, each node decides whether it should be a CH or not. For this, the nodes relies on an internal process with only local information.

One of the most important and referenced stochastic algorithm is Low-Energy Adaptive Clustering Hierarchy or LEACH [4]. LEACH is also a distributed method in which nodes convert themselves into CH if a probability value (*p*), is lower than a random value generated by the nodes internally. That threshold value is computed in each round, based on a simple equation of *p*. This probability increases with the number of rounds in which the nodes has not been selected as CH and that probability is reset only if the node is selected as CH. The probability reaches 1 after a constant number of rounds, which ensures that all the nodes become CHs within a given interval. This interval is based on the parameter *k*, which defines the number of desired CHs in the network. The value of *k* is defined in [4] as n∗p where *n* is the total number of nodes in the WSN and *p* is set to 0.05. This means that 5% of the nodes play the role of CH. There is also a centralized version by the same authors. This is called LEACH-C [8]. In LEACH-C, an artificial intelligence technique (simulating an annealing algorithm) is used to choose the optimal clusters from those with the highest energy. Another centralized technique is the one known as Base Station Controlled Dynamic Clustering Protocol (BCDCP) [9]. It is designed to have the same number of nodes attached to each CH. To do so, the CHs must communicate among them in some phases of the protocol. A different stochastic method is the one presented in [10], Multi-Threshold Long Lifetime Protocol (MTLLP), in which each node computes a probability threshold to be elected as a CH in a similar way that LEACH does. These thresholds are based in four variables related to the energy and distance. Nodes with a low energy level are not allowed to be CH.

One of the first clustering works that proposed a Fuzzy-I logic-based and centralised algorithm is described in [11]. Referred to as Cluster-head Election using Fuzzy Logic Algorithm (CHEF), it uses three input variables: energy, concentration and centrality. With a Fuzzy-I logic-based system, the output chance models the appropriateness of a node to become a CH. An alternative algorithm is Energy-Efficient Distributed Clustering based on Fuzzy (EEDCF) [12]. In this distributed algorithm, the close nodes coordinate to determine the CHs from the chances computed by each node. This computation is based on a Fuzzy-I logic-based system.

The proposal shown in [13], Type-2 Fuzzy Logic (Tyep2FL), classifies the nodes in different levels according to their distance to the BS. Then, the best cluster is chosen using a Type-2 fuzzy system. A centralized method that uses the Type-2 Fuzzy logic is analysed in [14], named Clustering Routing Protocol for WSN Based on Type-2 Fuzzy Logic and Ant Colony Optimization (CRT2FLACO). These techniques show that the use of the Type-2 Fuzzy logic improves the performance when compared with Type-I fuzzy logic systems. This improvement is due to the best management of the existing uncertainty, which is intrinsically present in this kind of network, as it is shown in the Cluster Head Enhanced Election Type-2 fuzzy Algorithm (CHEETAH) [15] or Enhanced Unequal Distributed Type-2 Fuzzy Clustering Algorithm (EUDCF) in [5].

Hybrid clustering algorithms are also possible. In this kind of algorithm, both stochastic and fuzzy logic systems are employed to elect the CHs in the network. In this way, Fuzzy-based Hyper Round Policy Clustering Algorithm [16] starts choosing the CH in a stochastic way (based on residual energy) and later it uses a fuzzy system that establishes how many rounds each node is going to be as CH in a distributed way.

From the studied bibliography, we have verified that centralized methods outperform the distributed ones. However, the works defining the centralized methods do not address how the information that the BS uses for the clustering is obtained. In particular, they commonly rely on residual energy and precise locations of the nodes. This implies that the nodes should be equipped with complex hardware (e.g., GPS), which could be a strong limitation in some WSN applications such as those in indoor infrastructures, subterranean environments or dense forests. Moreover, even in those applications where GPS is possible, its inclusion supposes an increment in the cost. In addition, most of the discussed papers do not provide a complete description about how some parameters (like coverage radius, transmission power of the advice message, etc.) are computed without inferring in additional control messages. We also concluded that Type-2 fuzzy systems are able to work with estimations better than Type-1 system. These estimations are necessary to reduce the control messages in real implementations.

In this paper we present a detailed description of a novel centralized method without incurring in additional hardware costs for the nodes. The Base Station decides, based on a Type-2 fuzzy system with data gathered from the wireless sensor network, which nodes are going to be CHs while setting an optimised skip. In contrast to the previous works, this method introduces a new scoring method for selecting CHs in a WSN based on a Type-2 Fuzzy system. it was designed to allow that the same elected CHs can keep in their role for several rounds. That behaviour is based on the inertia of those types of networks and it is supposed, as was stated in the hypothesis H1, that several skipped rounds greater than 2 can prolong the network lifetime. In addition, due to the high uncertainty observed in this kind of problem, we propose the use of a Type-2 fuzzy system whose output interval can give further versatility and adaptability to different networks layouts. The description about how the input parameters of the fuzzy system are computed is included in the next Section.

## 3. Proposed Method

The proposed method is a centralized algorithm that combines the decision about the CHs and the skip setting. The selection of the CHs is made by the identification of the best scores obtained from the inferred results of a fuzzy system. The BS runs this system applied to each node. The inputs for each fuzzy system are parameters that the base station gathers or estimates from the information obtained from the network. The output score ranges from 0 to 1. Thus, the BS manages the network in a rounds-based schedule that updates the score of the nodes after a variable interval of rounds called skip. The exact round scheduling is detailed in Section 3.2.

As previously commented, the way that the BS sorts the nodes to elect the CHs is based on the output of a Type-2 Fuzzy inference system (T2FIS) or score. Precisely, a high value implies a high probability of converting a node into a CH. Finally, the BS only selects *k* nodes with the highest value to be CH and informs all the nodes of their role: cluster head or contributing node. The T2FIS is explained in the next section. The parameter *k* is set in the configuration process.

Concerning the skip configuration, this parameter is set in the configuration phase of the WSN. The goal of this setting is obtaining a good performance of the network in terms of reliability (making all the measurements from all the sensors available as long as possible) and durability (keeping the functionality of most sensors).

### 3.1. Node Scoring

The fuzzy system used to infer a score for each node is a Type-2 one. This kind of artificial intelligence is especially suitable for WSN, in which there is no mathematical function to model its performance and in which the uncertainty degree is really high. Type-2 Fuzzy sets also work better in those applications whose input variables also have uncertainty because they could be estimations from measurements with a high level of tolerance. In WSN, it is necessary to rely on estimations to decrease the control information exchanged among the nodes and, as a consequence, the consumption due to the control tasks is jointly decremented. Type-2 Fuzzy systems are also applied in other technological fields such as decision in Mobile Ad hoc Networks [17], risk analysis in Marine Power [18], wireless indoor location [19] and PID control [20].

**Figure 2:**
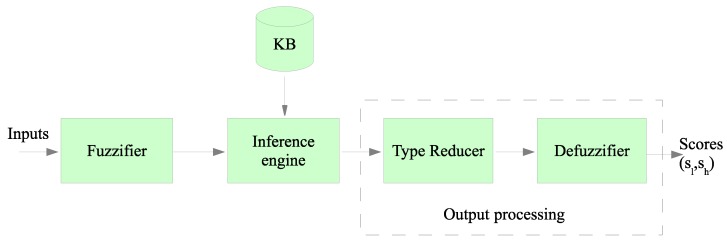
Block diagram of Type-2 fuzzy logic (T2FIS).

The block diagram of a Type-2 Fuzzy inference system is shown in Figure 2. As can be noticed, Type-2 Fuzzy systems differ from Type-1 systems only in the output processing block. Each input is fuzzified before entering into the inference system, that is connected to the knowledge base. The knowledge base includes the rules that link input and output variables to be used by the output processor. This last block differs from the Type-1, because the reducer is added before the final defuzzification is accomplished. Besides, the output of the T2FIS is an interval ($s_{l}$,$s_{h}$) instead of a crisp value obtained by a Type-I fuzzy system. Both limits of the interval are used when computing the best CHs according to the following rule: if the node was elected as CH in the previous round, then the lowest value of the interval, $s_{l}$, is chosen for a comparison operation. Otherwise, the highest value of the interval, $s_{h}$, is used in the comparison. This strategy was followed in some previous works such as [5,15]. This rule avoids that a node with a high output value could be selected continuously as a CH. This setting should be avoided as it would eventually deplete its battery.

The input variables of the T2FIS was carefully selected to achieve an efficient balance between energy consumption, performance and reliability (availability of the measurements from all the sensors in the network). Therefore, the input variables captures the evolution of the energy spent in every node and the behaviour of each node. Consequently, the T2FIS was designed with the following input variables:

- Residual Energy ($R_{i}$) of node *i*, measured as the ratio between the current energy and the initial energy of each node. The current energy of a node is estimated by the BS basing on the energy consumption model explained in Section 3.3.
- Normalized Distance ($D_{i}$), computed as the distance from the BS to the node *i* divided into the maximum one.
- The degree of deviation of the energy ($DDE_{i}$) in a node *i* ($R_{i}$) compared with the average energy of all the alive nodes ($R_{a}$). As the input variable of the fuzzy system must be kept between 0 and 1, this ratio is normalized according to Equation (1). Nodes with a high residual energy charge could saturate this value. For the normalization of $DDE_{i}$, the value 1.5 was set because it is assumed that a node with more than the 50% of the average energy should be a good candidate to be elected as CH and it is desirable to avoid higher levels of unbalanced battery charges in the network.
  (1) $$DDE_{i}=\left\{\begin{array}{cc} 1 & if\;R_{i}\geq 1.5\times R_{a}\\ \frac{R_{i}}{1.5R_{a}} & if\;R_{i}<1.5\times R_{a} \end{array}\right.$$
- Normalized number of rounds without being a CH ($RnoCH_{i}$). It is defined as the number of rounds without being CH ($roundsnoCH_{i}$) compared with a threshold ($RnoCH_{th}$). This input is established according to Equation (2).
  (2) $$RnoCH_{i}=\left\{\begin{array}{cc} 1 & if\;roundsnoCH_{i}\geq RnoCH_{th}\\ \frac{roundsnoCH_{i}}{RnoCH_{th}} & if\;roundsnoCH_{i}<RnoCH_{th} \end{array}\right.$$
  where $roundsnoCH_{i}$ is zero when the node *i* is elected as a CH and is incremented by one in each round in the opposite case.

This set of input variables makes the T2FIS exportable to a wide range of applications because they are application independent. Other parameter such as the on duty or idle periods or the rate of event detection could be considered in the configuration phase but without altering the T2FIS. As it will be discussed in the next section, the duration of a round could be adjusted for this purpose.

**Figure 3:**
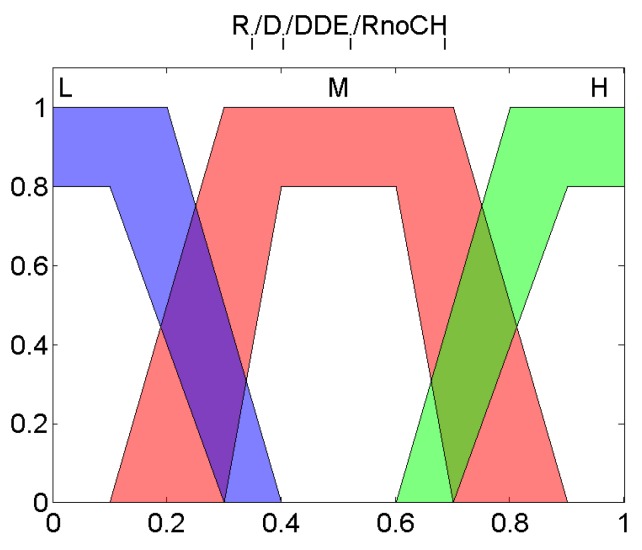
Membership functions of interval type-2 fuzzy system.

These variables, all of them normalized between 0 and 1, are the input of the fuzzy system depicted in Figure 2. Each of these variables are fuzzified with the Type-2 member function layouts that are depicted in Figure 3.

**Figure 4:**
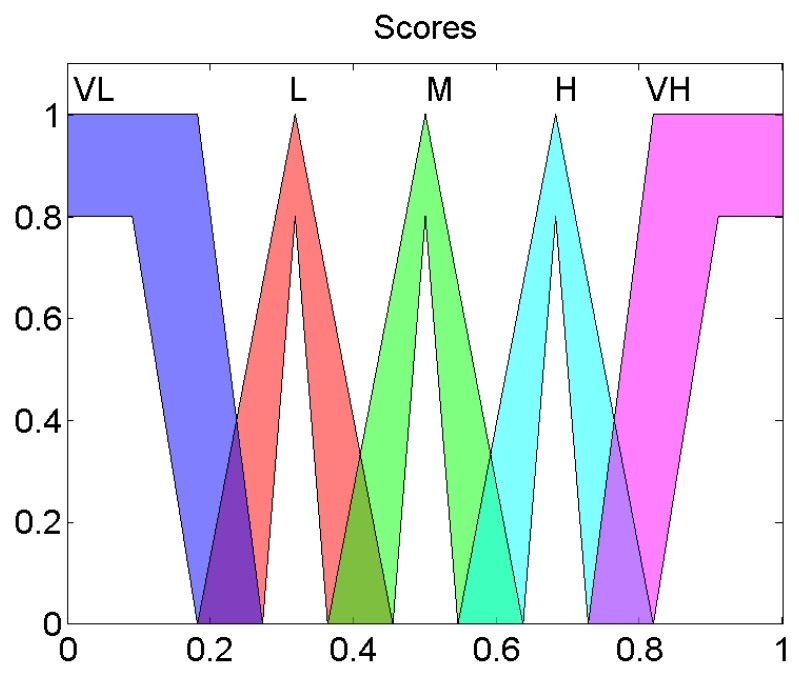
Output Membership functions of interval Type-2 fuzzy system.

Once they are fuzzified, the values will interrelate between them through the knowledge base that contains the IT-THEN rules of the expert system. Each rule is defined as follows: IF $R_{i}$ is $Value_{1}$ and $D_{i}$ is $Value_{2}$ and $DDE_{i}$ is $Value_{3}$ and $RnoCH_{i}$ is $Value_{4}$, THEN $score_{i}$ is *Output Value*, where $Values_{1}$, $Value_{2}$, $Value_{3}$ and $Value_{4}$ belong to low,medium,high as shown in Figure 3. The *Output Value* belongs to very low (VL), low (L), medium (M), high (H), very high (VH) as illustrated in Figure 4.

The rule base designed for the proposed method (see Table 1) is composed of 81 rules and, as can be observed, the effect of the qualitative value of each variable in the output score is different. For instance, high values of distance variable $D_{i}$ affect more negatively in the score than low values of residual energy $R_{i}$ due to the higher cost of long range communications compared with the threat of a low battery charge. Consequently, if rule 1 (L L L L -> L) is compared with rule 73 (H H L L -> VL), the output scores differ even when they share the same values for $DDE_{i}$ and $RnoCH_{i}$. For rule 1, the output is L however being far from the BS is penalized as modelled by rule 73. In rule 73, the output score is VL because $D_{i}$ is also high (a node far from the BS) even if the node has an almost full battery ($R_{i}$ is high). In the case of input variable RnoCH, its purpose is trying to achieve a good score even in the worst conditions (low battery and long distances e.g., rules 12, 15 or 21) for nodes that have not been CH for several rounds, whereas $DDE_{i}$ tries to exclude nodes that are supposed to be bad CHs candidates when the average available energy in the network is still high (e.g., rules 25–27 or 79–80).

The fuzzy system is a Mamdani one, in which the output variable is also a Type-2 fuzzy set like in Figure 4.

In the last block of the T2FIS (see Figure 2), the defuzzification is made and the output interval for the score ($s_{l}$,$s_{h}$), is obtained. Then, the intervals obtained for each node are used by the BS to elect the CHs accordingly to the process outlined in the next section.

### 3.2. Cluster Head Election Algorithm

As previously commented, the BS rules the network in a round-based schedule to minimize the message exchange necessary to decide the CHs and, as a result, the energy consumption of the nodes is reduced.

The overall process has two main phases: an initial setup phase and an operation round-based phase. This rounds-based scheduling must be synchronized with the desired event detection or measurement rate in order to fullfil the application requirements. Hence, the duration of any round must be carefully set to effectively detect the events or measure the physical magnitude under study. However, the time chosen for a round does not impact on the proposed algorithm because the energy spent in idle periods or node measurement is taken into account by variables $R_{i}$ and $DDE_{i}$ respectively. Thus, the design of the algorithm allows that the WSN has an effective adaptation to the timing requirements.

**Figure 5:**
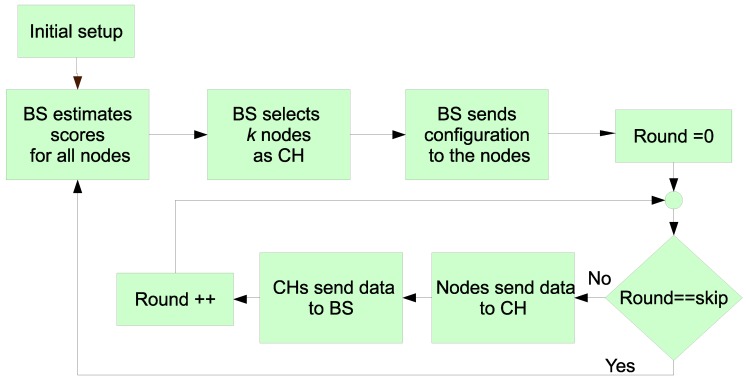
Proposed algorithm.

Once the initial setup is accomplished (see below for further details), the BS begins the operation phase. First, in order to elect the set of CHs for a round, it has to infer the scores for each node. Once it knows the scores for each node, it chooses the *k* nodes with the highest score to convert them into CH. To do so, it broadcasts that information to the network. The election of the next CHs is skipped a certain number of rounds (defined by the skip parameter), which is set by the BS in the initial phase. The different steps of the overall process are depicted in Figure 5 and detailed as follows.

Initial Setup

In the initial phase, the BS broadcasts a startup message to all the nodes in the covered area. Next, the nodes send to the BS a message at maximum power so that the BS candetermine the distance of each node to it. For this determination, the Received Signal Strength Indicator (RSSI) is used. Due to the method design, the exact position, and its corresponding hardware, is not needed as the system can work with an approximation thanks to the Type-2 fuzzy system. All the nodes hear this message and their replies so that they can compute the distances to the rest of the nodes too. In this way, in a future phase they can decide which node is the nearest CH.

CHs Selection

This is the first step of the operation phase. The BS computes the score value for all the nodes with the T2FIS. For previous CHs, the score corresponds to the lower limit in the output interval obtained by the T2FIS. Alternatively, for non-CHs, the score is the higher limit of the T2FIS output. With this selection of the score, we aim at assigning the role of CHs to most nodes during the complete WSN lifetime. In this way, a fair energy consumption is possible. Once the BS computes all the scores, it selects the best *k* nodes (according to their scores) to nominate them as CHs with a future message. The value of *k* indicates the optimal number of cluster heads. It was established in the 5% of the total number of nodes as was recommended in [4].

Cluster Configuration

The BS sends a broadcast message indicating which nodes are CH to all the nodes. With this message, the nodes join to the closest CH. The number of nodes in a cluster is not the same for all the structures, so the proposal is considered an unequal clustering method. The nodes joining a cluster adjusts their power transmission in order to just communicate with their corresponding CH.

Data Gathering

With this information, the rest of the nodes will send their information to the nearest CH. Each CH aggregates the data from the ascribed nodes, in such a way that it can also inform the BS if a node is not ascribed (death of a node). Additional information about the residual energy is also sent, so that the BS does not have to estimate those values for a future CH selection.

Skip Loop Iteration

In our proposal all the nodes are maintained as CHs during a skip number of rounds. In this phase, the BS updates a counter each time it receives information from the CHs. Once the counter equals the skip value, the BS steps on a new calculation of the scores of each node to restart the operation phase.

## 3.3. Computation of the Skip Value

The skip parameter tries to model the inertia of the network avoiding unnecessary CHs updates. In a previous work [5,15], we detected that the CHs election process tends to throw the same results from one round to the following one. Thus, it is commonly unnecessary to recompute the CHs. Consequently, we added the skip parameter to find a trade off between the consumption of the communications and the CHs actualization.

When determining the optimal value for the skip parameter, it is necessary to know the metrics used in wireless sensor networks, which are mainly referred to the lifetime of the network. The most used three parameters checked in the WSN analysis are (i) the round when the first node dies o First Node Dies (FND), (ii) the round when at least half of the nodes are death or Half Nodes Death (HND), and (iii) the round when the last node dies or Last Node Dies (LND). Depending on the application requirements, it will be of interest to favour one of these metrics or all of them. For instance, in the case of medical sensors the first death should be used, because a bad working of the network could imply lost measurements with severe consequences on the health’s patient. In other scenarios, like a WSN to measure/control the humidity of a terrain, the first death is not relevant because the WSN usually counts on with redundant sensors. If a high precision is not required, the other two performance metrics could be enough in this kind of application.

The computation of the optimal value for the skip parameteris going to be done through simulation. For this, the energy model to simulate data communications follows the first order radio model widely used in the related literature since its proposal in [4]. The model is described with Equations (3)–(5).

(3) $$E_{Tx}\left(l,d\right)=f\left(x\right)=\left\{\begin{array}{cc} l\cdot (E_{elec}+E_{fs}\cdot d^{2}), & d\leq d_{0}\\ l\cdot (E_{elec}+E_{mp}\cdot d^{4}), & d>d_{0} \end{array}\right.$$

(4) $$d_{0}=\sqrt{\frac{E_{fs}}{E_{mp}}}$$

(5) $$E_{Rx}\left(l\right)=E_{elec}\cdot l$$

where:

- *l* is the number of bits of any message.
- $E_{elec}$ the energy consumed in the transmitter or receiver circuitry for one bit.
- *d* is the distance that any message must cover.
- $E_{fs}$ is the energy consumed by the amplifier in free space model $\left(d\leq d_{0}\right)$ to get an acceptable bit error rate.
- $E_{mp}$ is the energy consumed by the amplifier in th multi-path (mp) model $\left(d\leq d_{0}\right)$ to obtain an acceptable bit error rate.

When a CH receives data from a contributing node, in addition to the energy spent in receiving the data, the CHs must spend energy in the data aggregation process. Therefore, the amount of energy spent in the reception process of *l*-bits is defined by Equation (6) for a CH:

(6) $$E_{Rx-DA}=\left(E_{elec}+E_{DA}\right)\cdot l$$

where $E_{DA}$ is the energy spent by the processing unit of a CH when aggregating the data received from a contributing node.

In a similar way to [4], the configuration values for the first order radio model used in Equations (3)–(6) are summarised in Table 2:

Two other configuration parameters are shown in Table 3. First, parameter *k* (the desired number of nodes to select by the BS) is calculated based on the estimation presented in [4], as it was previously commented in Section 2. Finally, parameter $RnoCH_{th}$ was set to 20 based on the value of *p* and taking into account the amount of nodes in the network. Thus it is assumed that a node should be elected as CH in no more than 20 consecutive rounds (see Equation (2)). In addition the length of the control and the data packets, $l_{control}$ and $l_{data}$, are 200 bits and 4000 bits respectively. These are common values in most of the research papers about clustering in WSN.

To effectively calculate the value of the parameter skip, it is necessary to estimate the energy spent by normal nodes and by the CHs during that period. Consequently, two equations were developed to model both processes. The energy consumed by a normal node (not elected as CH) during skip rounds is modelled by Equation (7):

(7) $$E_{sensor}=E_{Rx}\left(l_{control}\right)+E_{Tx}\left(l_{data},d\right)\times skip;$$

It includes the energy cost due to the reception of a control packet (in which the BS informs which nodes are CHs) and the energy cost of sending a data packet multiplied by the number of times that this process must be repeated (the value of the skip parameter). After the node joins a CH, it adjusts the transmission power according to the distance to the CH. In this case, we suppose that the distance *d* is small because normal nodes send the information to their closest CH, not to the BS.

The energy consumed by a node selected as CH is higher as can be noticed in Equation (8). To compute this consumption, we consider that the distance from the CH to the BS is usually greater than the distance to its contributing nodes.

(8) $$E_{CH}=E_{Rx}\left(l_{control}\right)+\left(E_{Rx-DA}\left(l_{data}\right)\times X+E_{Tx}\left(l_{data},d_{SB}\right)\right)\times skip;$$

Apart from the energy cost of the control message from the BS as in Equation (7), it includes the cost of reception and aggregation of *X* data packets from its contributing nodes, plus the message that must send to the BS each round with the aggregated data. It must be taken into account that the distance to the BS from the CH could be considerable, which converts that factor into the main energy cost.

**Figure 6:**
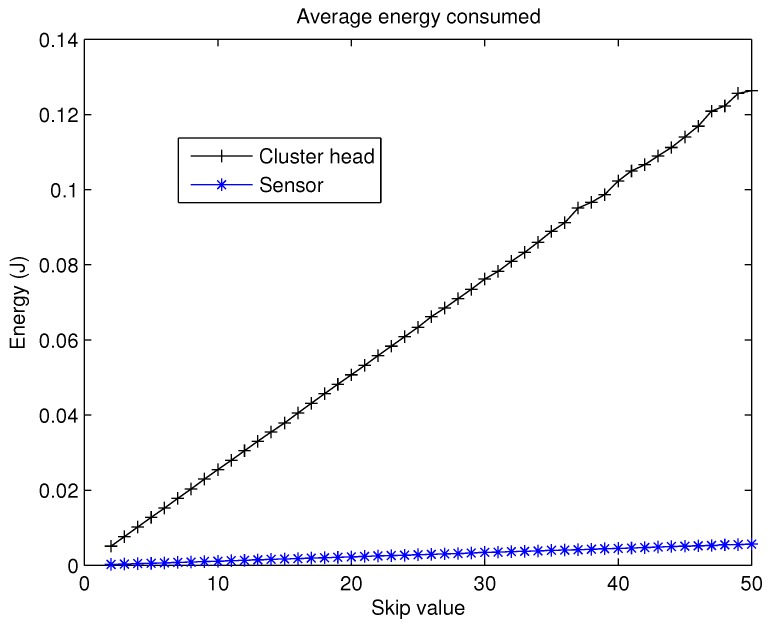
Energy consumed by CH and nodes.

It is evident that for high values of skip, a CH could spend enough energy to deplete its battery, even in the first rounds. This would eventually lead to a really low FND. Therefore, the present approach is based on the best balance between the number of CHs and the skip parameter because nodes that are not CH spend much less energy during skipped rounds. The evolution of the average energy spent by the nodes, acting as a normal node and as a CH can be studied in Figure 6. As can be noticed, the effect of being CH is clearly worse in terms of energy consumption when compared with a normal node. Consequently, the election of the value for the skip parameter is of paramount importance in CSBA.

As was commented above, to estimate the most appropriate value for the skip parameter, an empirical method, based on the simulation of three different scenarios of a wireless sensor network, was applied. All the scenarios are composed of 150 nodes randomly deployed over a deployment field of 100 m × 100 m and the initial energy of each node is set to 0.5 J. The difference among them is the location of the BS which was deployed as follows:

- Scenario 1. The BS is in position (100, 0) m, that means that it is in the border of the sensing area.
- Scenario 2. The BS is in position (150, 50) m, that means that it is outside the sensing area.
- Scenario 3. The BS is in position (50, 50) m, that means that it is in the centre of the sensing area.

**Figure 7:**
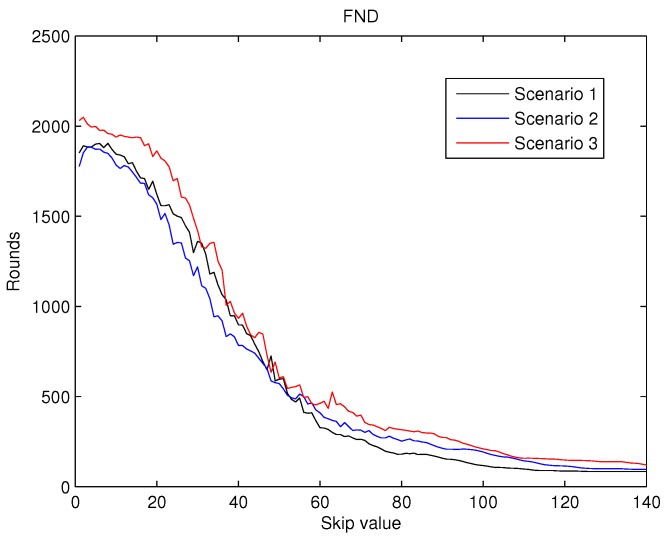
Round when the first node dies (FND) versus skip.

**Figure 8:**
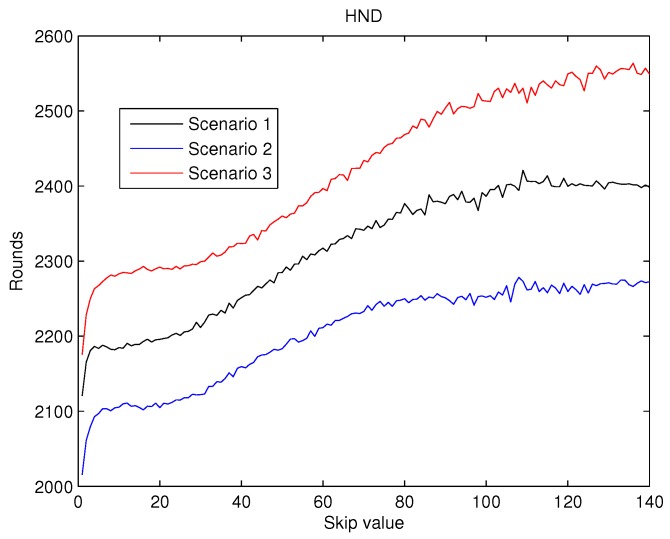
Round where half of the nodes are dead (HND) versus skip.

**Figure 9:**
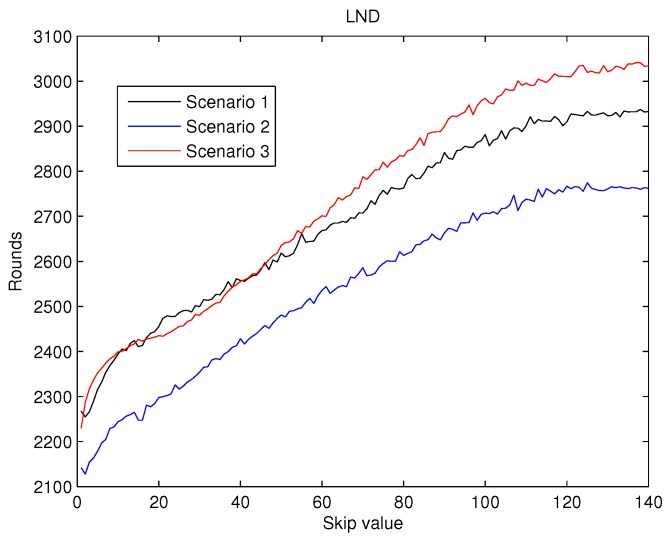
Round when at least 90% nodes are dead (LND) versus skip.

To achieve a statistically consistent result for the skip value a set of 30 different networks were simulated for any of the scenarios above. The average value of FND, HND and LND obtained for skip values from 0 to 140 are shown in Figure 7, Figure 8 and Figure 9.

The values for FND, shown in Figure 7 reveals that for the initial increments in the value of the skip, an improvement in this metric is achieved. Hence, this behaviour validates the first hypothesis exposed in the introduction. However, for higher values of the skip (over 10), this behaviour does not hold and the FND dramatically deteriorates. This second type of behaviour was also predicted by the second hypothesis mentioned previously.

The values obtained for HND can be seen in Figure 8. Here it can be observed that, when the skip value increases, HND values also increase, as predicted by the first hypothesis. This improvement is kept nearly constant when skip gets to 100 rounds. This behaviour is coherent with the second hypothesis with which we have been working.

The values obtained for LND are presented in Figure 9. LND increases almost linearly as the value of skip does, but when the skip gets to 100, LND is also kept almost unaltered. Consequently, the performance of the metrics confirms the hypothesis exposed in our introduction Section.

As a result, from the analysis of Figure 7, Figure 8 and Figure 9 it can be concluded that the effects of the increase of the skip value is inverse for FND than for HND and LND. This occurs because the energy cost of being CH is much higher than being a contributing node as is depicted in Figure 6. Thus, when the skip value is high, the CHs selected in the initial stages deplete their battery faster than any other node, which eventually drives to a premature FND value. However, if the CHs are selected more often (low values of skip), the balance of energy is better, but better nodes will die sooner than in other scenarios with higher skip values. Hence, a trade off between a high value of FND (reliability) and both HND and LND (network lifetime) is necessary in order to achieve the best performance of the network and its application.

Therefore, considering reliable applications to be deployed in the WSN, we take into account those three metrics when choosing the value of the skip parameter. Analysing the three metrics altogether, we focus on Figure 7, because FND is the most restrictive metric. From the simulations results, we set the skip parameter to 7 as the FND starts to deteriorate for higher values. The behaviour is similar for the three scenarios.

## 4. Results

The validity of the skip setting is verified with three different configurations related to BS positions corresponding to the scenarios described in the previous section. With these configurations, the algorithm is tested in the three modes commonly evaluated in the literature. The initial energy of each node is 0.5 J.

To check the performance of the proposed Centralized Skip Based Algorithm (CSBA), it is compared with other relevant proposals widely used in the literature. The evaluated algorithms are:

- A distributed stochastic algorithm referred to as LEACH [4]. In this well-known technique, the nodes have a probability of converting into CH. This probability increases with the number of rounds in which the nodes has not been selected as CH (see Section 2 for further detail).
- A centralized Type-2 fuzzy system algorithm known as Clustering Routing Protocol for Wireless Sensor Networks Based on Type-2 Fuzzy Logic and ACO (CRT2FLACO) [21]. The nodes that will be CH are selected with a fuzzy system in which the input variables are residual energy, distances to the BS and the number of neighbours. Later, the routers between CH are found using ACO to save energy sending data to the BS from the nodes.
- A centralized Type-1 fuzzy algorithm known as Cluster Head Election mechanism using Fuzzy logic (CHEF) [11]. A group of nodes is selected that are converted into CH from the output of the fuzzy system, that has residual energy, centrality and the number of near nodes, as inputs.
- A distributed Type-1 fuzzy system known as EEDCF [12]. In this case, the close nodes coordinate among them to decide the CH. For that operation, they exchange information about the output of the fuzzy system based on the residual energy, the number of close nodes and the average energy of those nodes. These last parameters constitute the inputs of the fuzzy system. The node with the higher output value becomes the CH for the close nodes.
- A distributed Type-2 Fuzzy system algorithm presented in [5]. The Enhanced Unequal Distributed Type-2 Fuzzy Clustering Algorithm (EUDCF) uses the residual energy, BS distance, the average number of nodes connected to the CH and the number of rounds without being CH as input variables.

These algorithms were implemented in Matlab according to their specifications described in the corresponding papers. The simulations are all run with the same energy model (see Equations (3)–(6)). In order to allow a statistically significant comparison, 30 simulations were made for each of the mentioned algorithms to obtain the average values for FND, HND and LND.

**Figure 10:**
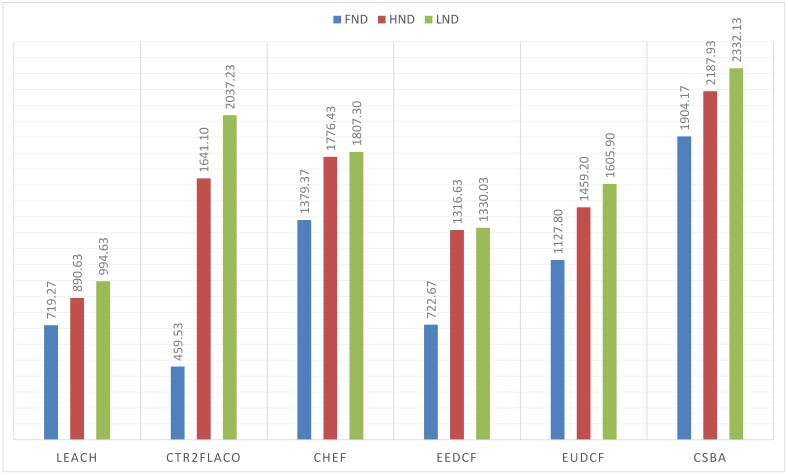
FND, HND and LND for 150 nodes in a 100 m × 100 m area with BS at (100, 0) m.

For the first set of simulations, the wireless sensor network corresponds to the Scenario 1. The results are shown in Figure 10. As it is observed, centralized methods get better metrics than the distributed ones. Moreover, the methods based on Type-2 fuzzy logic outperform their counterparts of Type-1. The results obtained by our proposal, CSBA, are the best in terms of FND, HND and LND when compared with all the other methods. Moreover, the result of FND in CSBA is greater than the LND value obtained with most methods. This implies that the network performance was improved in terms of reliability and durability when CSBA was applied.

**Figure 11:**
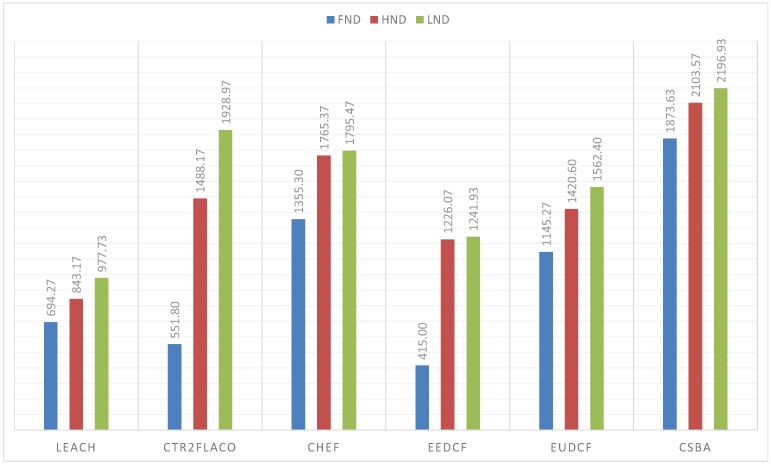
FND, HND and LND for 150 nodes in a 100 m × 100 m area with BS at (150, 50) m.

**Figure 12:**
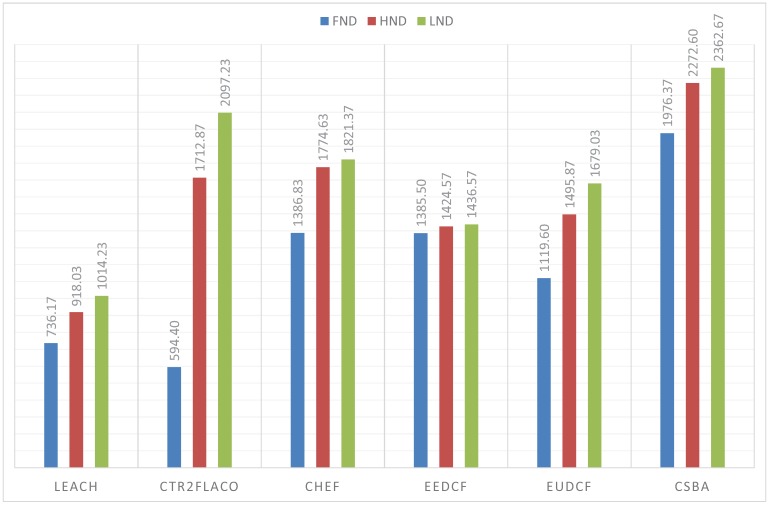
FND, HND and LND for 150 nodes in a 100 m × 100 m area with BS at (50, 50) m.

The simulations performed with the BS outside the covered area, that is, when the BS is at (150, 50) m, are in Figure 11. From these results, we can conclude that the centralized fuzzy systems outperfom the centralized ones. In addition, the Type-2 Fuzzy systems achieve a better performance than the type-I fuzzy systems. We can highlight that CSBA still gets the best results. A similar behaviour is reflected when the BS is at position (50, 50) m. The obtained metrics are in Figure 12.

## 5. Conclusions

In the present paper, we propose a new centralized clustering algorithm based on a Type-2 Fuzzy system. This method schedules the CH selection in a round manner, so that the control message exchange is reduced (executed every ’skip’ rounds) but the data transmission is not compromised. The most appropriate value for the skip parameter was judiciously determined according to the reliability and durability of the network. It was demonstrated that the proposed algorithm outperforms the other tested algorithms (including centralized and distributed methods). The results depicted in Figure 10, Figure 11 and Figure 12 also show that algorithms based on Type-2 Fuzzy system are more capable of adapting to different network layouts when compared with the algorithms that use Type-1 fuzzy systems. Moreover, the centralized clustering algorithms obtained better results than the distributed ones due to the common knowledge that the base station has about the whole network. However, as can be observed in Figure 7, Figure 8 and Figure 9 when achieving higher values of HND and LND with CSBA, FND will suffer a serious decrease. This will impact on the reliability of the network. The value chosen for the skip parameter may not suit other network layouts with a different amount of nodes or field dimensions (even when the selected skip value was valid for the three tested scenarios). In addition, CSBA does not take into account nodes which die during skip intervals. If those nodes are CHs, the reliability of the system could be seriously compromised and some information collected by those CHs could be lost.

Therefore, as future work, we will address the dynamic determination of the skip value with an additional intelligence-based system in order to achieve a better adaptability for different network layout, number of nodes and network dynamics. That new intelligent system, hosted in the BS, would monitor the evolution of the network (e.g., number of node deaths, average energy in network areas or concentration of alive nodes), to adapt the skip value in order to increase the network lifetime. Moreover, the BS could take into account events, such as the death of a CH during the skip interval or new thresholds and variables to elect CHs dynamically (even during a skip). From the behaviour observed in the input variables of the T2FIS, a new design of those fuzzy variables would be necessary in order to tune the design of the fuzzy sets and their fuzzification.

## Figures and Tables

**Table 1:** Rule base for the type-2 fuzzy controller.

Rule	$R_{i}$	$D_{i}$	${\mathrm{DDE}}_{i}$	${\mathrm{RnoCH}}_{i}$	Score		Rule	$R_{i}$	$D_{i}$	${\mathrm{DDE}}_{i}$	${\mathrm{RnoCH}}_{i}$	Score
1	L	L	L	L	L		42	M	M	M	H	H
2	L	L	L	M	L		43	M	M	H	L	M
3	L	L	L	H	M		44	M	M	H	M	H
4	L	L	M	L	M		45	M	M	H	H	H
5	L	L	M	M	M		46	M	H	L	L	L
6	L	L	M	H	L		47	M	H	L	M	L
7	L	L	H	L	L		48	M	H	L	H	L
8	L	L	H	M	M		49	M	H	M	L	VL
9	L	L	H	H	M		50	M	H	M	M	L
10	L	M	L	L	VL		51	M	H	M	H	M
11	L	M	L	M	VL		52	M	H	H	L	M
12	L	M	L	H	L		53	M	H	H	M	H
13	L	M	M	L	VL		54	M	H	H	H	VH
14	L	M	M	M	L		55	H	L	L	L	VL
15	L	M	M	H	L		56	H	L	L	M	VL
16	L	M	H	L	VL		57	H	L	L	H	L
17	L	M	H	M	L		58	H	L	M	L	VL
18	L	M	H	H	L		59	H	L	M	M	VL
19	L	H	L	L	VL		60	H	L	M	H	L
20	L	H	L	M	VL		61	H	L	H	L	VL
21	L	H	L	H	L		62	H	L	H	M	VL
22	L	H	M	L	L		63	H	L	H	H	L
23	L	H	M	M	L		64	H	M	L	L	M
24	L	H	M	H	L		65	H	M	L	M	M
25	L	H	H	L	L		66	H	M	L	H	H
26	L	H	H	M	L		67	H	M	M	L	M
27	L	H	H	H	M		68	H	M	M	M	M
28	M	L	L	L	L		69	H	M	M	H	H
29	M	L	L	M	M		70	H	M	H	L	M
30	M	L	L	H	H		71	H	M	H	M	H
31	M	L	M	L	M		72	H	M	H	H	VH
32	M	L	M	M	M		73	H	H	L	L	VL
33	M	L	M	H	H		74	H	H	L	M	L
34	M	L	H	L	M		75	H	H	L	H	L
35	M	L	H	M	H		76	H	H	M	L	L
36	M	L	H	H	VH		77	H	H	M	M	L
37	M	M	L	L	L		78	H	H	M	H	M
38	M	M	L	M	M		79	H	H	H	L	M
39	M	M	L	H	M		80	H	H	H	M	M
40	M	M	M	L	M		81	H	H	H	H	H
41	M	M	M	M	M							

Table key: VL = very low, L = Low, M = medium, H = high and VH = very high.

**Table 2:** Parameters for the losses in the first order radio model and data aggregation.

Parameter	Value
$E_{elec}$	50 nJ/bit
$E_{fs}$	10 pJ/bit/m2
$E_{mp}$	0.0013 pJ/bit/m4
$E_{DA}$	5 nJ/bit

**Table 3:** Parameter configuration for skip estimation.

Parameter	*k*	${\mathrm{RnoCH}}_{\mathrm{th}}$
Value	7 (150×p)	20 (1/p)

## References

[1] Cuevas-Martinez J.C., Gadeo-Martos M.A., Fernandez-Prieto J.A., Canada-Bago J., Yuste-Delgado A.J. (2010). Wireless Intelligent Sensors Management Application Protocol-WISMAP. Sensors.

[2] Liu X. (2012). A Survey on Clustering Routing Protocols in Wireless Sensor Networks. Sensors.

[3] Cenedese A., Luvisotto M., Michieletto G. (2017). Distributed Clustering Strategies in Industrial Wireless Sensor Networks. IEEE Trans. Ind. Inf..

[4] Heinzelman W.R., Chandrakasan A., Balakrishnan H. Energy-efficient communication protocol for wireless microsensor networks. Proceedings of the 33rd Annual Hawaii International Conference on System Sciences.

[5] Yuste-Delgado A.J., Cuevas-Martinez J.C., Triviño-Cabrera A. (2019). EUDFC - Enhanced Unequal Distributed Type-2 Fuzzy Clustering Algorithm. IEEE Sens. J..

[6] Singh S.P., Sharma S. (2015). A survey on cluster based routing protocols in wireless sensor networks. Procedia Comput. Sci..

[7] Afsar M.M., Tayarani-N M.H. (2014). Clustering in sensor networks: A literature survey. J. Netw. Comput. Appl..

[8] Heinzelman W.B., Chandrakasan A.P., Balakrishnan H. (2002). An application-specific protocol architecture for wireless microsensor networks. IEEE Trans. Wirel. Commun..

[9] Muruganathan S.D., Ma D.C.F., Bhasin R.I., Fapojuwo A.O. (2005). A centralized energy-efficient routing protocol for wireless sensor networks. IEEE Commun. Mag..

[10] Kia G., Hassanzadeh A. (2019). A multi-threshold long life time protocol with consistent performance for wireless sensor networks. AEU-Int. J. Electron. Commun..

[11] Indranil G., Riordan D., Srinivas S. Cluster-head election using fuzzy logic for wireless sensor networks. Proceedings of the 3rd Annual Communication Networks and Services Research Conference (CNSR’05).

[12] Zhang Y., Wang J., Han D., Wu H., Zhou R. (2017). Fuzzy-logic based distributed energy-efficient clustering algorithm for wireless sensor networks. Sensors.

[13] Nayak P., Vathasavai B. (2017). Energy Efficient Clustering Algorithm for Multi-Hop Wireless Sensor Network Using Type-2 Fuzzy Logic. IEEE Sens. J..

[14] Zhang Q., Sun Z., Zhang F. A clustering routing protocol for wireless sensor networks based on type-2 fuzzy logic and ACO. Proceedings of the 2014 IEEE International Conference on Fuzzy Systems (FUZZ-IEEE).

[15] Cuevas-Martinez J.C., Yuste-Delgado A.J., Triviño-Cabrera A. (2017). Cluster Head Enhanced Election Type-2 Fuzzy Algorithm for Wireless Sensor Networks. IEEE Commun. Lett..

[16] Neamatollahi P., Naghibzadeh M., Abrishami S. (2017). Fuzzy-Based Clustering-Task Scheduling for Lifetime Enhancement in Wireless Sensor Networks. IEEE Sens. J..

[17] Yuste A.J., Trivino A., Casilari E. (2013). Type-2 fuzzy decision support system to optimise MANET integration into infrastructure-based wireless systems. Expert Syst. Appl..

[18] Bahrebar S., Blaabjerg F., Wang H., Vafamand N., Khooban M.H., Rastayesh S., Zhou D. (2018). A novel type-2 fuzzy logic for improved risk analysis of proton exchange membrane fuel cells in marine power systems application. Energies.

[19] AL-Madani B., Orujov F., Maskeliūnas R., Damaševičius R., Venčkauskas A. (2019). Fuzzy Logic Type-2 Based Wireless Indoor Localization System for Navigation of Visually Impaired People in Buildings. Sensors.

[20] Kumar A., Kumar V. (2017). Evolving an interval type-2 fuzzy PID controller for the redundant robotic manipulator. Expert Syst. Appl..

[21] Xie W.X., Zhang Q.Y., Sun Z.M., Zhang F. (2015). A Clustering Routing Protocol for WSN Based on Type-2 Fuzzy Logic and Ant Colony Optimization. Wirel. Pers. Commun..

